# Resistance to cis- and carboplatin initiated by epigenetic changes in ovarian cancer patients

**DOI:** 10.20517/cdr.2019.010

**Published:** 2019-06-19

**Authors:** Heidi Schwarzenbach, Peter B. Gahan

**Affiliations:** ^1^Department of Tumor Biology, University Medical Center Hamburg-Eppendorf, Hamburg 20246, Germany.; ^2^Fondazione “Enrico Puccinelli” Onlus, Perugia 06126, Italy.

**Keywords:** Ovarian cancer, carboplatin, cisplatin, resistance, DNA methylation, histone modifications, microRNAs

## Abstract

Initially, most ovarian tumors respond to the treatment with platinum components, but frequently recurrence occurs within the following two years in advanced ovarian cancer patients. In this regard, previous studies have shown changes in the epigenetic patterns in ovarian cancer that are linked with resistance to cis- and carboplatin therapy. Thus, epigenetic changes mediated by a treatment with cis- or carboplatin could identify such patients who do or do not respond to this therapy. Therefore, an understanding of the impact of platinum on epigenetics in ovarian cancer is important in overcoming platinum resistance. In this review, we delineate epigenetic abnormalities in cis- and carboplatin-resistant ovarian tumors, such as changes in DNA methylation, histone modifications and deregulation of microRNAs, and discuss the potential of epigenetic therapies in combination with platinum.

## Introduction

Although ovarian cancer is a leading cause of gynaecological cancer deaths, the primary cause of the disease remains unclear. The lack of early markers of ovarian cancer and the development of drug resistance following chemotherapy with, e.g., platinum-based compounds retards attempts to better identify and treat this cancer.

In 1896, Baldwin postulated that individuals within a population with the “correct” allele could choose a new environment so resulting in a permanently changed evolutionary development within that environment^[1]^. It is now more than 60 years since Waddington^[2,3]^, following up this approach, introduced the term “epigenetics” to describe the concept that a characteristic acquired within a total population as a response to an environmental stimulus could be inherited in the absence of DNA mutations. For example phenotypic modifications can occur through the alteration of gene expression without any modification to the DNA sequence of the gene itself. Although the concept was not readily accepted at the time, epigenetics has subsequently become an important aspect of genetics and evolutionary theory, and is of particular interest in the study of cancer initiation, development and possible resolutions.

Specifically, epigenetic modifications involve DNA methylation, nucleosome repositioning, histone post-translational modifications and post-transcriptional gene regulation by miRNAs^[4]^. Histone modifications can affect chromatin structure resulting in the passage of heritable changes to the next generation. The role of histone epigenetic modifications in ovarian cancer has been comprehensively considered in a review by Yang *et al*.^[5]^. Although they have compiled an impressive listing of histone modifications, they considered that such studies are only at an early stage. Nevertheless, there are a number of epigenetic inhibitors being considered with protein modifying drugs already under clinical trials for ovarian cancers. Currently, there appears to be a low specificity for such compounds.

The major form of treatment for ovarian cancers, evolved from earlier studies on the use of platinum compounds inhibiting *Escherichia coli* cell division and solid tumors involves the use of platinum containing molecules^[6,7]^. A range of such compounds has been developed namely, cisplatin, carboplatin, oxaliplatin, nedaplatin and lobaplatin, the most commonly used for ovarian cancer treatment being cisplatin and carboplatin.

In the present review, we will examine DNA methylation and their involvement in the different forms of ovarian cancer together with the epigenetics of both histones and miRNAs and their possible roles concerning the reversal of resistance to cis- and carboplatin in ovarian cancer treatment.

## Characteristics of DNA methylation

Similarities between the early stages of normal embryological development and cancer development have been noted since before the mid 20th century. Epigenetic alterations involving DNA methylation can be considered as such an example, DNA methylation being a basic step essential to the early stages of embryogenesis. However, cancer initiation also involves alterations of DNA methylation in the silencing of tumor suppressor genes, the activation of oncogenes and the initiation of metastases. In particular, aberrant DNA methylation in cancer can be directly linked to drug resistance^[8]^.

DNA methylation normally occurs on the cytosine residues adjacent to a guanine residue (CG dinucleotides), the methyl group from S-adenosylmethionine being attached to cytosine by DNA methyltransferase. Thus, DNA modification involves 5-methylcytosine, 5-formylcytosine, 5-hydroxymethylcytosine and 5-carboxylcytosine^[9]^. The CG residues may be present singly along the DNA strands and tend to be constantly methylated. Alternatively, they may be present in clusters of 1000-2000 residues along the DNA in the form of CpG islands that are associated with gene promoters. Hence, if hypomethylated, the genes are active and if hypermethylated, the genes are silenced^[10]^. In many cancers, including ovarian cancer, a decrease in the global methylation of the heterochromatic chromosome regions results in the activation of a number of oncogenes, whilst the locus-specific hypermethylation of specific CpG island regions associated with promoters of tumor suppressor genes results in their inactivation^[4,11-13]^. Nevertheless, when methylation occurs, there are cases when only one allele maybe methylated and the other not. Hence, it depends on which allele is expressed as to the expression outcome, e.g., this can occur in ovarian cancer^[14,15]^. Gene expression by the non-methylated allele is likely for some genes so leading to continued expression despite the other allele being methylated. In their study, Losi *et al*.^[16]^ investigated 41 ovarian cancer associated promotor genes, and observed an intermediate level of hypermethylation (~50%) for most hypermethylated genes. Since there were > 70% of tumor cells present in each tumor sample employed, they would have expected either a high or a low methylation level. Combining their data with those described in the literature, where most studies dealt with very few genes, they proposed that this might be a general event of intra-tumoral heterogeneity existing for epigenetic changes.

Hypermethylation also has its effects with that of promoter CpG islands causing the silencing of tumor suppressor genes whilst methylation of CpG islands results in the inhibition of transcription factor suppressors.

Gene selection is also important for the study of ovarian cancer. Whilst Losi *et al*.^[16]^ and Choi *et al*.^[17]^ listed genes involved in cancer in general those epigenetically modifiable promoter genes relevant to ovarian cancer are given in Table 1. Genes involved in cisplatin/carboplatin resistance/sensitization of ovarian cancer are presented in Table 2.

**Table 1:** Epigenetically modifiable promoter genes relevant to ovarian cancer

	Promoter genes
DNA methylation	*APC*, *ESR*, *MGMT*, *RASSF1A*, *MLH1*, *TERT*, *WT1*
Testis/ovarian cells	*BORIS/CTCFL*, *DAX1*, *FOXL2*, *RSPO1*, *TMEFF2*
Wnt pathway	*APC*, *DKK1*, *DKK2*, *DKK3*, *SFRP1*, *SFRP4*, *SFRP5*, *WIF1*, *WNT4*
DNA repair pathways	*BRCA1*, *MGMT*, *MLH1*

APC: adenomatous polyposis coli; BORIS/CTCFL: brother of the regulator of imprinted sites/CCCTC-binding factor like; BRCA1: breast cancer 1; DAX1: dosage-sensitive sex reversal-adrenal hypoplasia congenital critical region on the X chromosome gene 1; DKK: dickkopf; ESR: estrogen receptor; FOXL2: forkhead box L2; MGMT: O-6-methylguanine-DNA methyltransferase; MLH1: mutL homolog 1; RASSF1A: ras association domain family member 1; RSPO1: R-spondin 1; SFRP: secreted frizzled-related protein; TERT: telomerase reverse transcriptase; TMEFF2: transmembrane protein with EGF like and two follistatin like domains 2; WIF1: WNT inhibitory factor 1; WNT4: Wnt family member 4; WT1: Wilms tumor 1

**Table 2:** Epigenetically modifiable genes relevant to ovarian cancer resistance and sensitization to cisplatin/carboplatin

Resistance/sensitization	Genes
Cisplatin resistance	*OXCT1*^[18]^, *GPCR*^[19]^, *TET1*^[20]^, *MLH1*^[21-23]^, *HOXA10, HOXA11*^[21,24]^, *NAGA*^[25]^, *UCHL1*^[26]^, *BCL2L1*^[27]^, *FANCF*^[28-30]^
Cisplatin sensitization	*FANCF*^[31]^, *NAGA*^[25]^, *CCDC69*^[32]^, *UCHL1*^[26]^
Carboplatin Resistance	*TMEM88*^[33]^, *DOK2*^[34,35]^, *p57(Kip2)*^[36]^, *Plk2*^[37]^, *HERV-K*^[38]^, *SFRP5*^[39]^, *SLFN11*^[40]^, *ASS1*^[41]^

ASS1: argininosuccinate synthase 1; BCL2L1: BCL2 like 1; CCDC69: coiled-coil domain containing 69; DOK2: docking protein 2; FANCF: fanconi anemia complementation group F; GPCR: protein coupled receptor; HOXA: homeobox A cluster; HERV-K: for HERV-K: human endogenous retrovirus type K; MLH1: mutL homolog 1; NAGA: N-acetylgalactosaminidase; OXCT1: 3-oxoacid CoA-transferase 1; Plk2: polo like kinase 2; SFRP5: secreted frizzled-related protein 5; SLFN11: schlafen family member 11; TET1: tet methylcytosine dioxygenase 1; TMEM88: transmembrane protein 88; UCHL1: ubiquitin C-terminal hydrolase L1

Finally, it appears to be necessary to consider the effect of the degree of methylation of each gene with respect to the histological type of ovarian cancer being investigated, e.g., epithelial, serous, endometrioid and mucinous since each may offer a different response depending upon the degree of methylation of a particular gene^[16]^.

### Methods for determining DNA methylation

When determining methylation levels, there are a number of approaches. The first consideration concerns tissue preparation before selection of that which is to be analysed. Thus, the tissue may be either histologically processed, often wax embedded or, alternatively, the tissue may be either as fixed or unfixed, frozen sections. The selected material to be analysed is isolated from the sections after microscopical analysis. Hence, there are three different factors involved relating to the tissue sample examined, namely, chemical fixation of the tissue, dewaxing of embedded material and the use of unfixed, frozen tissue.

Once the selected material has been removed from the sections, a relevant method for analysis of methylation levels needs to be selected. Some methods utilized for the detection of DNA methylation levels^[42-45]^: Digestion-based assay (PCR, qPCR, RT-PCR, cold PCR); High resolution melting luminometric methylation assay; HPLC-UV; Mass spectrometry; ELISA-based methods; Amplified fragment length polymorphism; Restriction fragment length polymorphism; Luminometric methylation assay; Pyrosequencing; Bisulfite sequencing; ELISA; Methylation ligation-dependent macro-array; High-throughput measurement technologies.

An in-depth analysis of the majority of the available methods was given by Olkhov- Olkhov-Mitsel & Bapat^[42]^ to detect methylated and hydroxyl-methylated DNA biomarkers. The methods are grouped under bisulphite-based strategies, restriction enzyme based methods and affinity-based strategies. Subsequently, Kurdyukov and Bullock^[43]^ developed a simple algorithm for selecting the most appropriate method for the material to be analyzed and for the identification of the form of methylation to be determined through either whole genome methylation profiling or identification of differentially-methylated regions or the methylation status of specific genes or digestion based assays or differentially-methylated loci or hydroxymethyl cytosine determination.

Clearly, given the range of methods available for the determination of different aspects of DNA methylation used by different authors, it becomes somewhat difficult to routinely compare results in the case of applied topics, such as ovarian cancer.

Using the various methods available, a number of abnormal DNA methylation patterns have been demonstrated in cancer cells with specific consequences being identified^[46]^. Hence, global hypomethylation can lead to chromosomal and genetic instability as well as reactivation of endoparasitic and repetitive genomic sequences. In addition, hypomethylation of gene bodies can activate incorrect sites of transcription initiation while the loss of promoter methylation can cause activation of metastasis and tumor promoting genes.

## Epithelial-mesenchymal transition

Epithelial-mesenchymal transition (EMT) is a hallmark of cancer progression and metastasis. During this process, epithelial cells go through phenotypic changes and acquire mesenchymal characteristics. They lose their cell polarity and cell-cell adhesion and acquire migratory and invasive properties, facilitating their migration through the extracellular matrix and settlement in other organs. This molecular reprogramming and cell switch lead to the loss of cytokeratins and epithelial-specific junction proteins, e.g., E-cadherin, mediated by upregulation of the transcriptional repressors Snail and Slug, ZEB1 and ZEB2, and Twist, and turning on the expression of mesenchymal markers e.g. Vimentin and N-cadherin. EMT is induced by a variety of signals, including the Wnt/β-catenin signaling pathway, Notch transcription factors, phosphoinositide-3 kinase (PI 3K)/Akt signaling^[47]^.

## Signalling pathways

The following paragraphs contain a short overview on the signaling pathways most frequently involved in platinum resistance.

### Wnt signaling

The Wnt signaling pathway is a complex developmental cell signaling pathway which plays an essential role in embryogenesis. The network is generally divided into the β-catenin dependent (canonical) and the β-catenin independent (non-canonical) pathways. Wnt proteins bind to receptors of the Frizzled and the low-density lipoprotein receptor-related protein families on the cell surface. Through several cytoplasmic components, the signal is transmitted to β-catenin which then enters the nucleus and forms a complex with the transcription factor TCF to activate transcription of Wnt target genes. The activation of the pathway leads to a variety of biological processes, including cell proliferation, differentiation and migration. Aberrant oncogenic activation of the Wnt signaling pathway is a common event in different cancer types. Main mechanisms by which Wnt signaling is dysregulated in cancer are mutations in β-catenin or other key pathway members, as well as hypermethylation and silencing of gatekeeper antagonists, such as the secreted frizzled-related protein (SFRP) and dickkopf (DKK), or overexpression of Wnt ligands or receptors, resulting in increased cancer cell proliferation and migration^[48]^.

### PI3K/Akt signaling

The activation of PI3K/Akt pathway regulates many different physiological processes, such as transcription, protein synthesis, metabolic responses and membrane trafficking, and specifically promotes growth and proliferation of adult stem cells. There are many factors that boost the PI3K/AKT pathway, including EGF, IGF-1 and insulin. Activation of PI3K phosphorylates and activates AKT, localizing it in the plasma membrane. AKT has diverse downstream effects, among others activating CREB, inhibiting p27 and activating mTOR (mammalian target of rapamycin). The pathway is antagonized by PTEN (phosphatase and tensin homologue). In many cancer types, this pathway is abnormally activated, resulting in reducing apoptosis and allowing proliferation^[49]^.

### Notch signaling

Depending on the cellular context, the Notch pathway regulates proliferation, differentiation and apoptosis. In adult tissues, Notch signaling is involved in tissue homeostasis and stem cell maintenance. The Notch signaling pathway also plays an established role in embryologic development and its deregulation is associated with diverse cancer types. It is activated by a receptor-ligand binding between two neighboring cells, leading to a conformational change of the Notch receptor. Following two cleavages, the Notch intracellular domain (NICD) is released into the cytoplasm. After translocation into the nucleus, NICD binds to ubiquitous transcription factor CSL and converts a large co-repressor complex into a transcription activating complex. The complex activates the transcription of Notch target genes, among others p21, cyclin D1 and 3, c-myc and members of NF-κB family^[50]^.

## Platinum compounds

**Figure 1:**
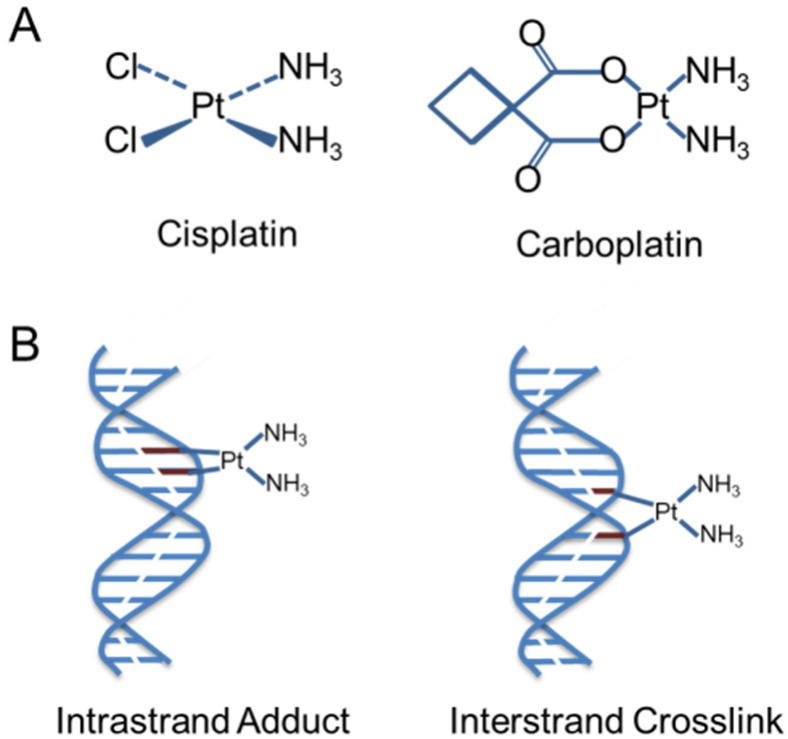
Chemical structures of cis- and carboplatin (A). Action of platinium. Cisplatin [Cis-diamminedichloroplatinum (II)] forms intracellular electrophilic water complexes based on the commonly predominant lower chloride concentrations. Due to its high affinity to the bases guanine and adenine, it forms chelates and inhibits DNA expression. Crosslinks of the DNA single and double strands are formed by platination with a disorder of template function and cell division. Carboplatin [Cis-Diamin (1,1cyclobutandicarboxylo)-platinium] has an equivalent mechanism of action. Chelation and cross-linking of the single and double-stranded DNA inhibit DNA synthesis and transcription triggering apoptosis (B)

The origin of platinum-cancer therapy dates back to the year 1965, when it was reported that cisplatin is an inhibitor of cell division in *Escherichia coli*^[6]^. In 1970, it was shown that cisplatin inhibits the growth of large tumors, possibly by inhibition of DNA synthesis^[7]^. Currently, cisplatin is one of the most effective chemotherapeutic agents and used for several tumor types. However, its clinical use is limited due to the severe side effects, including nephrotoxicity and acute kidney injury^[51]^. Carboplatin has been established as the successor to cisplatin with improved tolerability in many therapeutic regimens. This second generation analogue is closely related to cisplatin. Both cisplatin and carboplatin are the primary first-line therapies for the treatment of ovarian cancer. They are hydrolyzed in the cell, reacting with the sulfhydryl groups of proteins and nitrogen atoms of nucleic acids. Their covalent binding with purine bases introduces DNA damage, such as monoadducts or inter- and intra-strand crosslinks resulting in interference of the replication machinery, G2/M cell arrest and cell death by apoptosis or necrosis [Figure 1]. They can also induce oxidative stress by increasing mitochondrial reactive oxygen species and decreasing intracellular antioxidants, like reduced glutathione (GSH)^[52]^.

### Characteristics of platinum resistance

Acquired platinum resistance is considered as a multi-factorial process. Numerous mechanisms leading to the development of drug resistance have been reported and comprehensively considered^[53]^. They include e.g., changes in drug efflux through the modulation of diverse transporter systems, deregulated levels of intracellular glutathione and metallothioneine able to bind and sequester platinum, altered DNA repair pathways and reduced expression of pro-apoptotic proteins. These modulations are associated with epigenetic changes in DNA methylation, histone modifications and microRNA levels, but also with genetic alterations, such as mutations or deletion^[52,54]^. In this section, we present a short overview of the main mechanisms of cis- and carboplatin resistance to better illustrate the impact of these drugs on epigenetics in the following sections.

Cis- and carboplatin enter the cells and are exported from cells via transporters that e.g., manage copper homeostasis. The major copper influx transporter, copper transporter 1 (CTR1), controls the tumor cell accumulation and cytotoxic effect of cisplatin and carboplatin. Both copper and cisplatin may trigger the down-regulation of CTR1 via a process that involves ubiquitination and proteosomal degradation. In this regard, the majority of cells with acquired resistance to platinum drugs exhibit reduced drug accumulation. Thus, the cytotoxicity of these drugs correlates with the amounts of drugs entering the cell^[55,56]^.

Furthermore, oxidative stress is the one of most important mechanisms involved in cisplatin toxicity. Under normal physiological conditions, cells control reactive oxygen species levels by balancing the generation of reactive oxygen species with their elimination by e.g., GSH. Hence, glutathione acts as an antioxidant in the cell and supports the redox environment while conserving reduced sulfhydryl groups. Elevated levels of glutathione and glutathione-*S*-transferase, an enzyme mediating cisplatin coupling to GSH, induce resistance to cisplatin^[57]^. In this regard, cisplatin is detoxified by glutathione through adduct formation, and these platinum/glutathione conjugates are readily secreted out of the cells by e.g., multidrug resistance proteins of the ABC family^[58]^.

Finally, platinum damage is repaired primarily by the nucleotide excision repair system and the homologous recombination pathway. The nucleotide excision repair system recognizes platinum-induced inter- and intra-strand crosslinks and induces a process of DNA unwinding, incision, excision and synthesis. Induced DNA double-strand breaks are recognized by homologous recombination repair which initiates a process of single strand DNA formation, coating, filament formation, strand invasion and DNA synthesis. In particular, excision repair cross complementation group-1 and the related genes XPA and BRCA1 are involved in DNA repair. For example, 50% of high-grade serous ovarian cancers (HGSOC) exhibit defective DNA repair by inactivation of the homologous recombination due to germline and somatic mutations in BRCA1 (11%), BRCA2 (9%) and promoter hypermethylation of BRCA1 (10%). Homologous recombination deficiency may also result from PTEN homozygous loss, detected in about 7% of HGSOC^[59]^.

## DNA methylation

**Figure 2:**
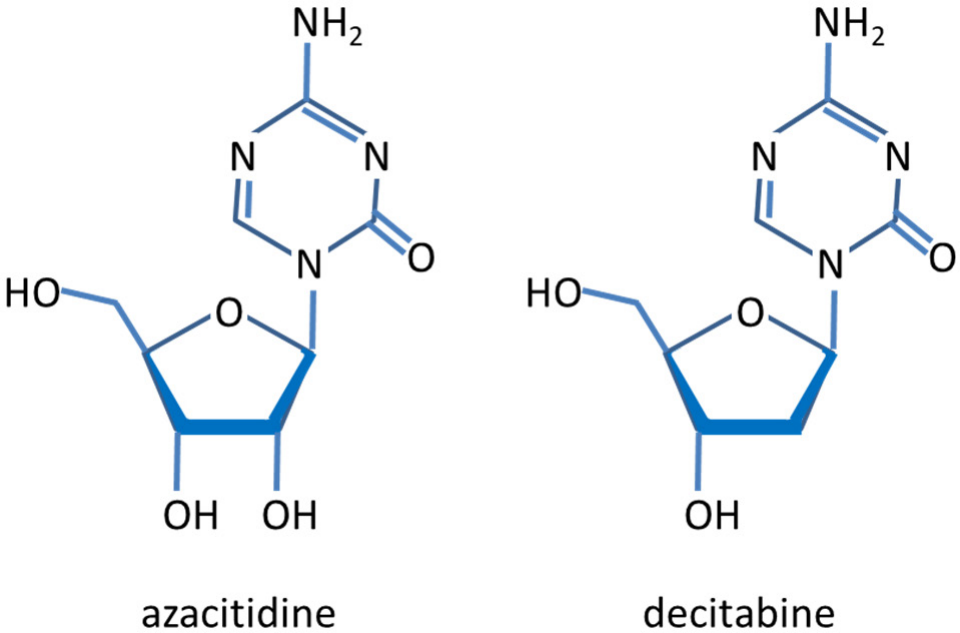
Chemical structures of azacitidine and decitabine. Azacitidine is metabolized intracellularly into decitabine

Epigenetic gene silencing is increasingly being recognized to contribute to the development of cis- and carboplatin resistance. Auspiciously, the treatment with demethylating agents has been shown to re-sensitize patients to platinum therapy demonstrating that DNA methylation is a critical factor in drug resistance. The most important DNA methyltransferase (DNMT) inhibitors are azacitidine and decitabine (5-aza-2′-deoxycytidine, dacogen). They are hypomethylating analogues of cytidine [Figure 2], and commonly used to treat myelodysplastic, hematological malignancies. Azacitidine was the first drug to demonstrate a survival benefit in a randomized trial for patients with myelodysplastic syndromes^[60,61]^. The following paragraphs give an overview on methylated genes that affect chemo-resistance.

### Cisplatin resistance

In ovarian cancer, selective epigenetic alterations of distinct biological pathways have been observed during development of platinum resistance. Hypermethylation-mediated repression of cell adhesion and tight junction pathways as well as hypomethylation-mediated activation of the cell growth-promoting pathways PI3K/Akt and TGF-β may contribute to platinum resistance^[62]^. As the following *in vitro* and *in vivo* studies demonstrate, chemo-resistance may be reversible by alteration of DNA methylation which may be an effective strategy to enhance the effectiveness of chemotherapeutic treatment in ovarian cancer.

High expression of DNMT1 is detected in S-phase of the cell cycle and makes DNMT1 a specific target for DNA methylation inhibition in rapidly dividing cancer cells. Covalent binding of DNMT1 by the nucleoside analogue SGI-110 results in DNMT1 proteolysis^[63]^. To assess the effects of SGI-110 on chemo-responsive genes silenced by DNA methylation in ovarian cancer, Fang *et al*.^[21]^ applied pyrosequencing. In vitro, they demonstrated that SGI-110 re-sensitized a range of cisplatin-resistant ovarian cancer cells, and induced significant demethylation and re-expression of tumor suppressor genes, differentiation-associated genes and even, putative drivers of ovarian cancer cisplatin resistance. *In vivo*, pyrosequencing of ovarian cancer xenografts confirmed that SGI-110 caused both global (LINE1 repetitive sequences) and gene-specific hypomethylation, including the tumor suppressor gene Ras Association Domain Family 1 (RASSF1A), the assumed drivers of ovarian cancer cisplatin resistance and the zinc finger protein ZIC1, the differentiation-associated genes HOXA10 and HOXA11 and the transcription factor STAT5B, as well as the DNA mismatch repair gene MLH1. The methylation of MLH1 in resistant cells has been investigated by several laboratories. Using genome-wide DNA methylation profiling, Zeller *et al*.^[22]^ identified genes becoming hypermethylated in chemo-resistant ovarian cancer cells. In particular, they found that MLH1 had a direct role in conferring cisplatin sensitivity when reintroduced into cells *in vitro*. These findings supported previous observations from Strathdee *et al*.^[23]^, 20 years ago. DNMT activity and thus, DNA methylation are regulated by the epidermal growth factor receptor (EGFR)^[64]^. Granados *et al*.^[65]^ examined whether cisplatin induces EGFR mediated changes in DNA methylation that are associated with the development of cisplatin resistance. Acute cisplatin treatment activated EGFR and downstream signaling pathways, as well as induced an EGFR-mediated increase in DNMT activity. This led to an increase in global DNA methylation in cisplatin resistant cells. During repeated cisplatin treatments, EGFR inhibition re-sensitized the cells to cisplatin and inhibited increases in DNA methylation and DNMT activity.

The ten-eleven translocation (TET) family of dioxygenases comprising TET1/2/3 is involved in DNA demethylation. Using cisplatin-sensitive and cisplatin-resistant ovarian cancer cell models, Han *et al*.^[20]^ detected that TET1 was significantly upregulated in cisplatin-resistant ovarian cancer cells compared with cisplatin-sensitive cells. Its ectopic cell expression promoted cisplatin resistance and decreased cytotoxicity induced by cisplatin via active DNA demethylation of vimentin, resulting in partial EMT.

Applying Illumina human methylation arrays and Affymetrix arrays, Bonito *et al*.^[31]^ showed that CpG sites within the homeobox transcription factor MSX1 gene had significantly lower levels of methylation in high-grade serous epithelial ovarian cancer patients who recurred by 6 months than patients who recurred after 12 months. In cisplatin-resistant ovarian cancer cell lines, MSX1 overexpression led to cisplatin sensitization, increased apoptosis and increased cisplatin-induced expression of the cyclin-dependent kinase (CDK) inhibitor p21. A further CDK inhibitor of cell cycle progression is p27, also known as KIP1. Zhao *et al*.^[66]^ detected that the expression level of p27 was dramatically downregulated in chemo-resistant cells, but treatment with the demethylating agent 5-aza-2'-deoxycytidine restored p27 expression in cisplatin resistant cells and increased sensitivity to cisplatin. Overexpression of p27 arrested the cell in the S phase and promoted an apoptotic response to cisplatin.

To analyze genome-wide DNA methylation profiles of cisplatin sensitive and resistant ovarian cancer cell lines, Yu *et al*.^[27]^ applied methyl-Capture sequencing (MethylCap-seq), which combines precipitation of methylated DNA by the recombinant methyl-CpG binding domain of MBD2 protein with next generation sequencing (NGS). They found a lower global CpG methylation in resistant cells. Methylation-specific PCR and bisulfite sequencing confirmed hypermethylation of protein tyrosine kinase 6, protein kinase Cε and the antiapoptotic gene BCL2L1 in sensitive cells compared with resistant cells. Performing genome-wide analyses of hypermethylated CpG islands in combination with real-time PCR, Kritsch *et al*.^[67]^ identified Tribbles 2 (TRIB2) as the most pronounced downregulated gene on mRNA level among 37 commonly epigenetically silenced genes in cisplatin-resistant ovarian cancer cells. Its re-expression increased the sensitivity to cisplatin and other DNA-damaging agents in these cells, whereas its knockdown increased the resistance to cisplatin in sensitive cells. TRIB2, that belongs to the family of pseudokinase proteins and degrades the myeloid transcription factor CCAAT enhancer binding protein α^[68]^, induced a cisplatin-dependent cell cycle arrest and apoptosis by acting on p21 and survivin expression. It seems to be involved in the signal transduction from nucleotide excision repair of intrastrand cross links. In line with its downregulation in ovarian cancer cells, tumors from cisplatin-resistant patients also expressed the lowest levels of TRIB2^[67]^.

Using an integrated approach of analyzing simultaneously gene expression levels and DNA methylation profiles, Yang *et al*.^[18]^ analyzed mRNA expression on gene chip arrays and DNA methylation profiles on methylation bead chips. Among 26 genes that were differentially expressed and methylated between cisplatin-resistant and -sensitive ovarian cancer cells, 3-oxoacid CoA transferase 1 (OXCT1) was selected for further investigations. OXCT1, that catalyzes the first and rate-determining step of ketolysis^[69]^, was hypermethylated at CpG sites of its promoter and downregulated in cisplatin-resistant cells. Treatment with a DNMT inhibitor restored hypermethylation-mediated gene silencing of OXCT1 in cisplatin-resistant cells, but not in cisplatin-sensitive cells. Notably, overexpression of OXCT1 conferred sensitivity to cisplatin in the ovarian cancer cells^[18]^.

The gene encoding for myelin and lymphocyte protein (MAL) has been reported to be among the most highly expressed genes in serous ovarian cancers from short-term survivors (< 3 years) compared with those of long-term survivors (> 7 years)^[70]^. Lee *et al*.^[71]^ showed that this difference in MAL expression is due to differences in DNA methylation at specific sites within the MAL promoter. MAL was largely unmethylated at the transcriptional start site in serous ovarian cancers. Methylation of the region 200-400 bp upstream of the promoter could be reduced by the treatment of an ovarian cancer cell line with 5-azacytidine, resulting in a 10-fold increase in MAL expression. MAL transcript levels were also higher in cisplatin resistant ovarian cell lines suggesting that MAL methylation status serves as a marker of platinum sensitivity.

α-N-acetylgalactosaminidase (NAGA) is responsible for deglycosylating the group-specific component (Gc), a precursor of Gc protein-derived macrophage activating factor (GcMAF). The deglycosylated form of Gc protein cannot be converted into GcMAF and subsequently, decreased GcMAF levels can promote immunosuppression^[72]^. Ha *et al*.^[25]^ identified NAGA as one of the key candidate genes for cisplatin drug response. In cisplatin-resistant cell lines, NAGA was significantly downregulated and hypermethylated at its promoter. Restoration and overexpression of NAGA in cisplatin-resistant lines induced cytotoxicity in response to cisplatin, whereas depletion of NAGA increased cisplatin resistance^[25]^.

In their study, Cui *et al*.^[32]^ investigated coiled coil domain containing protein 69 (CCDC69), and found that its inhibition may interfere with the effectiveness of a combination therapy with platinum drugs. The expression levels of CCDC69 were 3-4 fold higher in cisplatin-resistant cells than its parental cisplatin-sensitive cells. Treatment of CCDC69 knockout, cisplatin-resistant cells with cisplatin was accompanied with increasing sensitivity to cisplatin, abrogation of G1 and G2/M arrest, increasing caspase activity, p53 acetylation and higher levels, as well as mitochondrial redistribution of the apoptosis modulator Bax.

Ubiquitin carboxyl terminal hydrolase 1 (UCHL1) catalyzes the hydrolysis of COOH-terminal ubiquityl esters and amides^[73]^. Jin *et al*.^[26]^ detected UCHL1 promoter methylation in ovarian cancer cell lines and a negative correlation of UCHL1 with their cisplatin resistance. Microarray data revealed that after UCHL1 knockdown several apoptosis related genes, including apoptosis regulators BCL2, BCL11A, AEN and XIAP, and the phosphorylated serine/threonine protein kinase AKT were up-regulated, whereas the pro-apoptotic Bax was down-regulated.

FANCF (Fanconi anemia, complementation group M) is a gene associated with Fanconi anemia, the protein products of which were reported to interact with proteins involved in DNA repair pathways, e.g., with BRCA1^[74]^. More than 15 years ago, Olopade and Wei^[28]^ described a model of ovarian cancer tumor progression that implicated aberrant FANCF promoter methylation and correlated with gene silencing and disruption of the Fanconi-anemia-BRCA pathway. Disruption of this pathway occurred de novo in ovarian cancer and might contribute to selective sensitivity to platinum components. The laboratory of D’Andrea^[29]^ investigated the relationship of chromosome instability with cisplatin hypersensitivity, and showed that the phenotype of ovarian cancer cells was caused by methylation and silencing of the signaling Fanconi-anemia-BRCA pathway. Restoration of this pathway was associated with demethylation of FANCF, leading to acquired cisplatin resistance. The laboratory proposed a model for ovarian tumor progression in which the initial methylation of FANCF was followed by FANCF demethylation, ultimately resulting in cisplatin resistance^[30]^.

AT-101 is a natural compound from cotton seeds and inhibits the anti-apoptotic Bcl-2 family of proteins^[75]^. Karaca *et al*.^[76]^ investigated the effects of AT-101 in combination with cisplatin on the expression of pro-apoptotic proteins and epigenetic events in ovarian cancer cells. Combined administration of both agents led to a strong synergistic cytotoxicity and apoptosis in human ovarian cancer cells and reduced among others Bcl-2 and inhibited both DNMT and histone deacetylase (HDAC) activities.

Applying reduced representation bisulfite sequencing, Lund *et al*.^[19]^ examined malignant ascites cells from patients with high-grade serous ovarian cancer, the most common ovarian cancer type, to clarify the molecular mechanisms of drug resistance in this cancer type. Cisplatin resistance was associated with hypomethylation at several CpG sites, primarily localized in the intergenic regions of the genome. The genes close to the differentially methylated sites were associated with canonical pathways, such as cAMP-mediated signaling, G-protein coupled receptor (GPCR) signaling, WNT/beta-catenin signaling and human embryonic stem cell pluripotency.

### Carboplatin resistance

Several mechanisms associated with the development of acquired drug resistance in ovarian cancer have also been reported for carboplatin. Fang *et al*.^[35]^ compared the response of patients with recurrent platinum-resistant ovarian cancer who received carboplatin plus the DNMT inhibitor guadecitabine with a standard-of-care chemotherapy regimen. Epigenomic and transcriptomic profiling were performed using the Infinium methylation bead chips. They defined 94 gene promoters that were significantly hypomethylated by guadecitabine, with 1,659 genes differentially expressed in pretreatment versus post-treatment tumors resulting in altered immune reactivation and DNA repair pathways. In functional analyses, upregulation of the tumor suppressors docking protein 2 (DOK2), an adapter protein downstream of tyrosine kinase, and miR-193a silenced by promoter methylation restored platinum drug sensitivity of ovarian cancer cells. Lum *et al*.^[34]^ also characterized and functionally validated DOK2 among the genes identified in the epigenome screening using a tissue culture carboplatin resistance assay. In the set of methylated candidate genes associated with platinum resistance, the loss of DOK2 induced chemotherapy resistance by decreasing the level of apoptosis in response to the treatment. de Leon *et al*.^[33]^ used an Illumina DNA methylation array and profiled carboplatin sensitive and resistant ovarian cancer xenografts. In particular, they confirmed that the mRNA expression levels of transmembrane protein 88 (TMEM88) were increased in resistant compared to control xenografts and correlated with promoter hypomethylation. Its transcriptional regulation by promoter methylation was supported by the administration of ovarian cancer cells with the DNMT inhibitor guadecitabine which increased TMEM88 mRNA expression levels. TMEM88 knock-down re-sensitized cells to platinum and induced upregulation of cyclin D1 and c-Myc, suggesting that TMEM88 inhibited the Wnt signaling pathway.

In approximately 11% of high-grade serous ovarian cancer, BRCA1 promoter methylation is an important somatic driver^[77]^. Using whole-genome sequencing of tumor and germline DNA samples from these patients, Patch *et al*.^[78]^ observed several molecular events associated with acquired resistance to carboplatin-combination treatment. They identified a patient who displayed extensive promoter methylation and low expression of BRCA1. During the relapse, the patient lost BRCA1 methylation and the gene was expressed at comparable levels to homologous-recombination-intact tumors. Comparison of the global methylation patterns of primary and recurrence samples suggested a specific rather than a generalized altered methylation status at relapse in this patient.

A phase 1 trial of low-dose decitabine combined with carboplatin in ten patients with recurrent, platinum-resistant ovarian cancer was performed by Fang *et al*.^[79]^ The most common toxicities were nausea, allergic reactions, neutropenia, fatigue, anorexia, vomiting and abdominal pain. LINE-1 hypomethylation in peripheral blood mononuclear cells (PBMCs) and hypermethylation of HOXA11 and BRCA1 in plasma were detected. One complete response was observed, and three additional patients had stable disease for about six months. Furthermore, a phase 1b-2a clinical trial of a sequential combination of azacitidine and carboplatin was initiated in patients with platinum-resistant or platinum-refractory epithelial ovarian cancer by Fu *et al*.^[80]^ Among 29 evaluable patients, this treatment produced one complete response, three partial responses and ten cases of stable disease. The predominant toxicities were fatigue and myelosuppression. Correlative studies indicated that DNA methylation of the human leukocyte antigen death receptor 4 (DR4) in peripheral blood leukocytes was decreased during treatment in three of four objective responders, but in only five of 13 non-responders. In a phase 2 clinical trial, Matei *et al*.^[24]^ tested the clinical and biological activity of decitabine administered before carboplatin in platinum-resistant ovarian cancer patients. Low-dose decitabine altered DNA methylation along with the Wnt signaling and apoptosis pathways, restored the sensitivity to carboplatin in patients with heavily pretreated ovarian cancer and resulted in a high response rate and prolonged progression-free survival (PFS). Demethylation of the DNA mismatch repair gene MLH1, the tumor suppressor genes RASSF1A, HOXA10 and HOXA11 in tumors positively correlated with PFS.

Using an ovarian cancer cell line with acquired resistance to carboplatin and genome-wide microarray profiling, Coley *et al*.^[36]^ identified the CDK inhibitor p57 (Kip2) to be downregulated in carboplatin resistance. Methylation sites in the p57 promoter and even, a preferential sensitivity to seliciclib, a CDK inhibitor, were detected in the cell line. Silencing of p57 decreased the apoptotic response to the effects of platinum, but unexpectedly produced sensitization to seliciclib. High levels of p57 mRNA in tumor biopsies correlated with complete responses to chemotherapy and improved outcome.

The serine proteases urokinase plasminogen activator and tissue-type plasminogen activator together with their major physiological inhibitor, plasminogen activator inhibitor-1 [PAI-1; serine protease inhibitor clade E member 1 (SERPINE1)] have been identified as prognostic factors for disease progression and relapse in different cancer types since they play important roles in cell adhesion, migration and invasion^[81]^. Recently, Pan *et al*.^[82]^ revealed that that SERPINE1 may be a promising therapeutic target for chemo-resistance of ovarian cancer cells. Microarray screening showed that carboplatin treatment caused hypomethylation of the promoter of SERPINE1, and consequently, significantly increased the expression of SERPINE1, resulting in induction of the EMT process with decreased expression of E-cadherin and increased expression of Vimentin, Snail and Twist.

As reported by Syed *et al*.^[37]^, DNA methylation of the Polo-like kinase Plk2 in tumor tissues and serum samples was associated with a higher risk of relapse in patients treated postoperatively with carboplatin and paclitaxel. They found that platinum resistance can be conferred by the downregulation of Plk2 transcripts via promotor methylation in ovarian cancer cells selected for paclitaxel and carboplatin resistance, primary tumors and patient sera. In the drug-resistant cells, Plk2 promoter methylation varied with the degree of drug resistance and transcriptional silencing of the promoter. Knockdown of Plk2 abrogated G2-M cell-cycle blockade by paclitaxel, conferring resistance to both paclitaxel and platinum. Contrary, ectopic expression of Plk2 restored sensitivity to G2-M cell cycle blockade and cytotoxicity triggered by paclitaxel.

Furthermore, Iramaneerat *et al*.^[38]^ demonstrated that the expression levels of human endogenous retrovirus (HERV) K and E were increased in tissues from patients with ovarian clear cell carcinoma (OCCC). Methylation levels of HERV were associated with treatment response and prognosis of OCCC. DNA methylation levels of HERV-K, HERV-E and LINE-1 were decreased in tissues from patients with advanced stage cancer. In particular, HERV-K was significantly less methylated in the platinum-resistant cohort. Hypomethylation of HERV-K correlated with a shorter overall and progression-free survival.

Apart from genetic events, epigenetic modification of the SFRP family has been shown to be important in regulating the Wnt signaling pathway^[48]^. Su *et al*.^[39]^ demonstrated that restoration of SFRP5 expression attenuated Wnt signaling in ovarian cancer cells. Cancer cell growth, invasion of cells and tumorigenicity were inhibited in mice independently of the canonical pathway. Epigenetic silencing of SFRP5 led to oncogenic activation of the Wnt pathway and contributed to ovarian cancer progression and carboplatin resistance through the transcription factor Twist-mediated EMT and AKT2 signaling.

Finally, Glasspool *et al*.^[83]^ also tested the hypothesis whether a DNA hypomethylating agent can reverse resistance to carboplatin in women with relapsed ovarian cancer. Surprisingly, and in contrast to SGI-110, the administration of decitabine appeared to reduce rather than increase the efficacy of carboplatin in partially platinum-sensitive ovarian cancer patients. These findings provoked the authors to suggest that other demethylating agents should be considered in future combination studies.

### Cis- and carboplatin resistance

To date, there are some studies that have compared the resistance to both, cis- and carboplatin. Using a comprehensive DNA methylation microarray platform, Nogales *et al*.^[40]^ investigated the relationship of resistance to both platinum compounds with the DNA methylation of the putative DNA/RNA helicase Schlafen-11 (SLFN11). They identified hypermethylation of promoter CpG sites associated with the silencing of this gene that correlated with increased resistance to cis- and carboplatin. Notably, their clinical findings showed that those ovarian cancer patients harboring epigenetic inactivation of SLFN11 had a poor response to both drugs.

In the biosynthesis of arginine, argininosuccinate synthetase (ASS1) is the rate-limiting enzyme^[84]^. Down-regulation of its expression was associated with the development of platinum resistance in ovarian cancer^[85]^. Nicholson *et al*.^[41]^ showed that ASS1 expression correlated with the ability of ovarian cancer cells to grow in media supplemented with cisplatin, carboplatin or taxol or in arginine-depleted media. Aberrant methylation of the ASS1 promoter correlated with transcriptional silencing in ovarian cancer cell lines leading to selective resistance to platinum-based drugs and conferred arginine auxotrophy and sensitivity to arginine deprivation. In ovarian cancer patients at diagnosis, ASS1 methylation was associated with significantly reduced overall survival and relapse-free survival. In patients who relapsed, ASS1 methylation was significantly more frequent compared to patients who did not relapse, suggesting that hypermethylated ASS1 contributes to treatment failure in ovarian cancer.

Metalloestrogens are metals that activate the estrogen receptor in the absence of estradiol. They encompass two subclasses: metal/metalloid anions and bivalent cationic metals. Arsenite and selenite belong to the subclass of metal/metalloid anions^[86]^. Aebi *et al*.^[87]^ demonstrated that selection of cells for resistance to platinum resulted in resistance to arsenite and selenite. Since mammalian cells detoxify arsenite and selenite by S-adenosylmethionine dependent methylation, they examined whether the latter is involved in the cellular metabolism of cisplatin. Treatment of ovarian cancer cells and their cisplatin-resistant subline with the S-adenosylhomocysteine hydrolase inhibitor adenosine-dialdehyde, an indirect inhibitor of transmethylation, led to a significant increase in the cellular content of S-adenosylhomocysteine without changing S-adenosylmethionine. Adenosine dialdehyde synergistically enhanced the cytotoxicity of both, cisplatin and carboplatin. These findings indicate that inhibition of S-adenosylmethionine dependent transmethylation enhanced the toxicity of cisplatin and carboplatin in ovarian cancer cells *in vitro* without directly affecting the metabolism of either platinum drug.

## Histone modifications

Epigenetic modifications of histones include methylation, acetylation, phosphorylation, sumoylation, glycosylation, ubiquitination, carbonylation and ADP-ribosylation of individual histone components^[88]^. Such histone modifications have a direct effect upon DNA through nucleohistone repositioning. Histone changes are heritable and can result in modification of gene expression. Furthermore, the epigenetic modifications of histones lead to the deregulation of miRNAs in tumor cells^[89]^. The effect of epigenetic changes in ovarian cancer is well-reviewed by Yang *et al*.^[5]^

The combination of DNA methylation inhibitors and HDAC inhibitors synergistically activates gene expression^[90,91]^. Thus, not only DNA methylation but also histone deacetylation has a central role in the transcriptional repression of tumor suppressor genes and genes involved in sensitivity to chemotherapy^[91]^.

### Cisplatin resistance

There are a few articles on the relationship of histone modifications to platinum resistance. In this regard, Cacan *et al*.^[92]^ identified HDAC and DNMT1 to exhibit an aberrant association with the regulator of G protein signaling 10 (RGS10) in chemoresistant ovarian cancer cells. Knockdown of HDAC1 or DNMT1 expression and pharmacological inhibition of DNMT or HDAC enzymatic activity significantly increased RGS10 expression and cisplatin-mediated cell death. Moreover, DNMT1 knockdown decreased HDAC1 binding to the RGS10 promoter in chemo-resistant cells, suggesting HDAC1 recruitment to RGS10 promoters requires DNMT1 activity.

In both *in vitro* (cisplatin-resistant ovarian cancer cells) and *in vivo* (xenografts), Steele *et al*.^[93]^ observed that the combination of decitabine and a clinically relevant inhibitor of HDAC activity (belinostat) increased the expression of epigenetically silenced MLH1 gene and MAGE-A1 antigen when compared with decitabine alone. The treatment that influenced the histone structure improved the efficacy of chemotherapy in tumors that had acquired drug resistance due to DNA methylation and gene silencing. Liu *et al*.^[94]^ also performed *in vitro* and *in vivo* studies. In cisplatin-resistant ovarian cancer cells, they showed that HDAC1 knockdown suppressed cell proliferation and increased apoptosis. The increase in chemo-sensitivity was caused by downregulating the oncogene c-Myc and upregulating miR-34a. Cisplatin treatment activated HDAC1 and c-Myc and inactivated miR-34a. Inhibition of HDAC1 reduced c-Myc expression, increased miR-34a expression and sensitized ovarian cancer cells to cisplatin-induced apoptosis. *In vivo* studies confirmed these findings. They showed that targeting HDAC1 sensitized murine xenograft models to cisplatin treatment. Cacan^[95]^ demonstrated that expression of the death receptor FAS is suppressed in cisplatin resistant ovarian cells compared to parental cells. Surprisingly, no difference in DNA methylation was observed at FAS promoters between both cell lines. However, there were a decrease in acetylated histone H3 and a corresponding increase in HDAC1 associated with FAS promoter in resistant cells. Knockdown of HDAC1 and pharmacological inhibition of HDAC enzymatic activity significantly increased FAS expression in resistant cells, suggesting that particularly histone modifications may contribute to the loss of FAS expression in cisplatin resistant ovarian cancer cells, and that enhancement of FAS expression may increase tumor cell sensitivity to immune cells.

Histone modifications in chemo-resistant cells were evaluated in relationship to oncolytic adenovirus efficacy by Hulin-Curtis *et al*.^[96]^. In contrast to cisplatin-sensitive ovarian cells displaying an efficient shortening of cell viability by adenovirus in the presence of cisplatin, cisplatin-resistant cells diminished this reduction of cell viability mediated by adenovirus with increasing doses of cisplatin. HDAC2, and to a lesser extent HDAC1, were up-regulated in cisplatin-resistant but not in cisplatin-sensitive cells. Administration of cisplatin-resistant cells with trichostatin A (TSA), a HDAC inhibitor significantly enhanced adenovirus mediated reduction of cell viability in the presence of cisplatin. Cells treated with TSA alone did not display this effect, indicating an adenovirus dependent mechanism.

### Carboplatin resistance

In a phase I trial, Falchook *et al*.^[97]^ demonstrated that sequential treatment with a combination of the nucleoside analogue azacytidine, the HDAC valproic acid and carboplatin decreased DR4 methylation, but there was no relationship with either tumor response or number of therapy cycles received. A modest evidence of antitumor activity could only be observed in patients with heavily treated advanced malignancies.

## miRNAs

Besides DNA methylation, the dysregulation of microRNAs (miRNAs) may also be responsible for the induction of acquired platinum resistance in ovarian cancer. MiRNAs are, together with long non-coding RNAs (lncRNAs) and small RNAs, members of the non-coding RNAs (ncRNAs)^[98]^. Whilst lncRNAs have been confirmed to be epigenetically modified, it is only recently that miRNA epigenetic modifications have been identified and related to cancer^[99]^. MiRNAs are small ncRNAs that bind to 3’-untranslated regions of target mRNA in a sequence-specific fashion and either inhibit the translation of their target mRNA or degrade it. MiRNAs are involved in both normal and cancer cellular processes linked to cell division, growth, differentiation and ageing^[100]^. Their behavior is complex in that they are present in both, the nucleus and cytoplasm, and their nuclear presence permits them to control gene expression^[101,102]^. However, subgroups of miRNAs, e.g., deregulated epi-miRNAs present in different cancer types target specific epigenetic regulators, such as DNMT and histone deacetylase^[103,104]^.

A number of clinically regulated miRNAs have been identified in ovarian cancer. These include upregulated miRNAs, miR-15a/16 miR-20a, miR-23a/b, miR-30a/b/c, miR-92, miR-93, miR-106a, miR-146b, miR-182, miR-200, miR-203, miR-205, miR-223 and downregulated Let-7a/b/d/f, miR-22, miR-31, miR-34a/b/c, miR-125b, miR-127-3p, miR-152, miR-155, miR-181a-3p, miR-382^[105]^.

### Cisplatin resistance

A wealth of publications deals with the role of miRNAs in platinum resistance [Table 3]. For example, Vera *et al*.^[106]^ identified four miRNAs (miR-7, miR-132, miR-335 and miR-148a) the deregulation of which appears to be a common event in the development of resistance to cisplatin in ovarian cancer. In particular, the specific DNA methylation of miR-7 in cisplatin-resistant cell lines was associated with a poor prognosis in ovarian cancer patients. The direct regulation of MAFG by miR-7 seems to cause this resistance.

**Table 3:** MiRNAs in cis- and carboplatin resistance

miRNAs	-regulation	Targets	Ref.
Cisplatin resistance			
miR-7	Down-	MAFG	[106]
let7e	Down-	BRCA1, Rad51	[108,109]
miR-9	De-	BRCA1	[110,111]
miR-21	Up-	NAV3, PTEN, c-IAP2, PDCD4	[112,147-149]
miR-23a	Up-	n.d.	[113]
miR-24-3p, miR-192-5p, miR-139-5p, miR-155-5p	Up- Up Down- Down-	MAPK signaling pathway BRCA1, RB1, CDK1, ABL1, CCNA1 MET, SHC1, EGFR, INPPL1 MET, SHC1, EGFR, INPPL1	[150]
miR-29	Down-	n.d.	[151]
miR-30a/c	Down-	DNMT1, Snail	[152]
miR-30a-5p, miR-34c-5p	Up-	n.d.	[153]
miR-31	Up-	KCNMA1	[114]
miR-34a	Down-	HDAC1	[115]
miR-93	Down-	DNA polymerase η	[154]
miR-101	Down-	EZH2	[155]
miR-106a	Up-/down-	PDCD4/MCL1	[156,157]
miR-125b	Up-	BAK1	[158]
miR-128	Down-	Bmi-1, ABCC5	[116]
miR-130a	Down-/up-	MDR1, PTEN, XIAP	[159-162]
miR-130b	Down-	CSF-1, MDR1, GST-π	[117,163]
miR-133b	Down-	MDR1, GST-π	[164]
miR-136	Down-	n.d.	[118]
miR-139-5p	Down-	c-Jun	[122]
miR-141	Up-	KEAP1	[124]
miR-142-5p	Down-	XIAP, BIRC3, BCL2, BCL2L2, MCL1	[165]
miR-149-5p	Up-	MST1, SAV1	[166]
miR-152	Down-	ERCC1	[167]
miR-152 miR-185	Down-	DNMT1	[168]
miR-186	Down-	MDR1 (ABCB1), GST-π, Twist1	[169,170]
miR-199a	Down-	DDR1, ITGB8, mTOR	[125,171,172]
miR-199b-5p	Down-	Jagged1	[173]
miR-204	Down-	IL-6 receptor	[174]
miR-224-5p	Up-	protein kinase C	[126]
miR-330-5p	Down-	S100A7	[175]
miR-335-5p	Down-	BCL2L2	[176]
miR-363	Down-	Snail	[177]
miR-376c	Up-	ALK7	[178]
miR-429	Down-	ZEB1	[179]
miR-449a	Down-	NOTCH1	[127]
miR-483-3p	Up-	protein kinase Cd	[128]
miR-489	Down-	Akt3	[180]
miR-490-3p	Down-	ABCC2	[181]
miR-497	Down-	mTOR, p70S6K1	[182]
MiR-509-3p	Down-	GOLPH3, WLS	[131]
miR-520g	Up-	DAPK2	[132]
miR-551b	Up-	FOXO3, TRIM31	[134]
miR-634	Down-	CCND1, GRB2, ERK2, RSK2	[183]
miR-708	Down-	IGF2BP1	[184]
miR-770-5p	Down-	ERCC2	[136]
miR-873	Down-	MDR1 (ABCB1)	[185]
miR-1294	Down-	IGF-1 receptor	[138]
Carboplatin resistance			
miR-146a	Down-	n.d.	[140]
miR-148b-5p	Down-	n.d.	[141]
miR-141, miR-200, miR-429	Down-/up-	n.d.	[186]
miR-484	DOwn-	VEGF B, VEGF receptor 2	[187]
Cis- and carboplatin resistance			
miR-21, miR-181a, miR-223, miR-486, miR-1908	Down-/up-	n.d.	[143]
miR-622	Up-	Ku complex	[146]

ABCC2: adenosine triphosphate-binding cassette subfamily C member 2; ABCC5: ATP-binding cassette subfamily C member 5; ALK7: activin receptor-like kinase 7; BAK1: Bcl-2 antagonist killer 1; BCL2: B-cell lymphoma-2; BCL2L2: BCL2 like 2; BIRC3: baculoviral IAP repeat-containing 3; CDK1: cyclin-dependent kinase 1; CCNA1/CCND1: cyclin A1/D1 gene; CSF-1: colony-stimulating factor 1; DAPK2: death-associated protein kinase 2; DDR1: Discoidin Domain Receptor 1; DNMT: DNA methyltransferase; EGFR: epithelial growth factor receptor; ERCC1: excision repair cross-complementation group 1; ERK1: extracellular signal-regulated kinase 1; EZH2, enhancer of zeste homolog 2; FOXO3: forkhead box O3; GRB2: growth factor receptor-bound protein 2; GST-π: glutathione S-transferase π; GOLPH3: Golgi phosphoprotein-3; IAP2: inhibitor of apoptosis protein-2; IGF2BP1: insulin-like growth factor 2 mRNA-binding protein 1; INPPL1: inositol polyphosphate phosphatase-like 1; ITGB8: integrin subunit beta 8; KEAP1: Kelch-like erythroid-derived cap-n-collar homology- (ECH-) associated protein-1; KCNMA1: potassium channel calcium activated large conductance subfamily M alpha, member 1; MFAG: musculoaponeurotic fibrosarcoma oncogene family, protein G; MCL1: myeloid cell leukemia sequence 1; MDR1: multidrug resistance 1; MET: mesenchymal-epithelial transition factor; MST1: STE20-like kinase; PDCD4: programmed cell death 4; PTEN: phosphatase and tensin homolog; mTOR: mammalian target of rapamycin; NAV3: neuron navigator; RB1: retinoblastoma 1; RSK2: ribosomal protein S6 kinase; S100A7: S100 calcium-binding protein A7; SAV1: salvador homolog 1; SHC1: Src Homology 2 Domain Containing 1; TRIM31: ring finger, B-box and coiled-coil domain protein, tripartite motif; VEGF: vascular epithelial growth factor; WLS: wntless (Wnt) ligand secretion mediator; XIAP: X-linked inhibitor of apoptosis; ZEB1: zinc finger E-box binding homeobox 1; n.d.: not determined

The human let-7 family comprises 13 members located on nine different chromosomes. The majority of the members is involved in the modulation of drug sensitivity in different cancer types^[107]^. In epithelial ovarian cancer, the laboratory of Wang demonstrated that deregulation of let-7e promoted the development of resistance to cisplatin^[108]^. In situ hybridization revealed significantly lower expression levels of let-7e in chemo-resistant than chemo-sensitive ovarian cancer tissues. Transfection with let-7e sensitized ovarian cancer cells to cisplatin, down-regulated BRCA1 and Rad51 expression and repressed the repair of cisplatin-induced DNA double strand break. Low let-7e and high Rad51 levels were significantly associated with poor overall and progression-free survival. Multivariate regression and receiver operating characteristic analyses showed that let-7e was an independent predictor for chemotherapy response and highly predictive of resistance to cisplatin, suggesting that re-expression of let-7e may be an effective strategy for overcoming chemo-resistance^[109]^. Zhao *et al*.^[110]^ revealed that primary cancer cells from drug sensitive patients are more tumorigenic than those from drug resistant women. In 26 drug-sensitive patients, the expression levels of miR-9, miR-145 and miR-429 were higher than in 20 drug-resistant cases. Conversely, higher miR-26a expression was observed in resistant patients. Inhibition of miR-9 resulted in decreased clonal cell formation and sensitivity to cisplatin, while knockdown of the other three miRNAs did not influence drug sensitivity. Sun *et al*.^[111]^ analyzed the effects of miR-9 on cisplatin and PARP [Poly(ADP ribose) polymerase] inhibitor sensitivity in ovarian cancer cells and xenograft mice. The impact of miR-9 on prognosis was assessed in a cohort of 113 ovarian cancer patients. In ovarian cancer cells, miR-9 bound directly to the 3’-UTR of BRCA1, downregulated BRCA1 expression and impeded DNA damage repair. Treatment with miR-9 sensitized BRCA1-proficient xenograft tumors to cisplatin. Patients with higher levels of miR-9 had better chemotherapy responses, platinum sensitivity and longer progression-free survival.

Using microarrays, Pink *et al*.^[112]^ identified miR-21-3p, the passenger strand of the known oncogenic miR-21, to direct increased resistance to cisplatin in a range of ovarian cell lines, whereas miR-21-5p had an opposite effect and increased cisplatin sensitivity. The induction of resistance to cisplatin by miR-21-3p may be caused by the binding to its mRNA target of the neuron navigator NAV3. NAV3 is involved in axon guidance during development and its knockdown increased resistance to cisplatin.

Jin *et al*.^[113]^ showed that inhibition of miR-23a expression could significantly increase the sensitivity to cisplatin in ovarian cancer cells. The cells were arrested in G0/G1 phase along with an increased apoptosis rate. In addition, the expression levels of P-glycoprotein involved in multi-drug resistance (MDR) decreased with increasing cisplatin concentrations.

Using microarrays and RNA-sequencing, Samuel *et al*.^[114]^ assessed the role of miR-31 in the development of chemo-resistance to cisplatin. They found increased levels of miR-31 and reduced levels of potassium channel calcium activated large conductance subfamily M alpha, member 1 (KCNMA1), a subunit of calcium-regulated big potassium (BK) channels in resistant ovarian cells. Overexpression of miR-31, knockdown of KCNMA1 or inhibition of BK channels increased resistance to cisplatin, suggesting that this resistance was mediated by the repression of KCNMA1 through miR-31.

Recently, Lv *et al*.^[115]^ showed that the overexpression of HDAC1 decreased cisplatin sensitivity, promoted proliferation and blocked the suppressive effects of miR-34a on cell proliferation in ovarian cancer cells. Accordingly, miR-34a directly bound to HDAC1, and downregulated its expression, which subsequently decreased the resistance to cisplatin and suppressed proliferation in ovarian cancer cells.

In both epithelial ovarian cancer cell lines and ovarian carcinomas, Li *et al*.^[116]^ analyzed the expression of miR-128 and its targeted genes, the polycomb ring finger oncogene Bmi-1 and the ATP-binding cassette subfamily C member 5 (ABCC5). MiR-128 expression was significantly reduced in the cisplatin-resistant ovarian cancer cell line compared with its parental SKOV3 cells, and decreased upon treatment with cisplatin in a concentration-dependent manner. Overexpression of miR-128 re-sensitized the cells to cisplatin and reduced the expression of cisplatin-resistant-related proteins ABCC5 and Bmi-1. Administration of a combination of cisplatin and miR-128 inhibited the growth of cisplatin resistant xenograft tumors more effectively than cisplatin alone.

Investigating the role of miR-130b in the development of multidrug-resistance, Yang *et al*.^[117]^ detected that down-regulation of miR-130b in ovarian cancer correlated with FIGO III-IV clinical stages, poor histological differentiation and its hypermethylation. Demethylation by the treatment with 5-aza-CdR re-activated miR-130b expression in drug resistant ovarian cancer cell lines along with an increase in sensitivity to cisplatin and taxol. Thus, downregulation of miR-130b promotes the development of multidrug resistant ovarian cancer partially by binding of miR-130b to its target mRNA of the colony-stimulating factor 1 (CSF-1).

As reported by Zhao *et al*.^[118]^, miR-136 expression was significantly reduced in 34 primary platinum-resistant patients and an ovarian cancer cell line. Overexpression of miR-136 decreased the chemo-resistance to cisplatin in ovarian cancer cells through inhibition of cell survival and promoting an apoptotic response to cisplatin. The percentage of DNA in comet tails, tail length, tail moment and olive tail moment exposed the relevance of miR-136 in the repair of cisplatin-induced DNA damage.

MiRNA-139 has been characterized as a tumor suppressor with anti-oncogenic and anti-metastatic activity and consequently, is downregulated in different cancer types^[119-121]^. Jiang *et al*.^[122]^ revealed that the expression of miR-139-5p was decreased in cisplatin-resistant ovarian cancer cell lines. Re-expression of miR-139-5p increased the sensitivity of these cells to cisplatin treatment, inhibited the expression of the activator protein-1 transcription factor component c-Jun through binding to the 3´UTR of c-Jun mRNA, and decreased the expression of the BCL family member BCL-xL, promoting cisplatin-induced mitochondrial apoptosis.

Oxidative and electrophilic changes in cells are mainly coordinated by the KEAP1/NRF2 [Kelch-like erythroid-derived cap-n-collar homology- (ECH-) associated protein-1/nuclear factor (erythroid-derived 2)-like 2] axis. Electrophiles react with critical thiol groups of KEAP1 causing the loss of its ability to inhibit NRF2. The KEAP1/NRF2 signaling pathway also down-regulates NF-κB transcriptional activity and attenuates cytokine-mediated induction of pro-inflammatory genes^[123]^. van Jaarsveld *et al*.^[124]^ demonstrated that miR-141 directly targets KEAP1, and that downregulation of KEAP1 induced cisplatin resistance. Conversely, overexpression of KEAP1 significantly enhanced cisplatin sensitivity. The NF-κB pathway, which is regulated by KEAP1, was activated upon miR-141 overexpression, while inhibition of this pathway partially reversed miR-141-mediated cisplatin resistance. Furthermore, van Jaarsveld *et al*.^[124]^ quantified the expression levels of miR-141 in 108 serous and 24 non-serous primary ovarian tumors. They found that its levels were elevated in non-serous ovarian tumors that did not respond well to therapy.

A negative correlation between the collagen receptor tyrosine kinase Discoidin Domain Receptor 1 (DDR1) and miR-199a-3p was detected by Deng *et al*.^[125]^ in ovarian cancer tissues. Cell culture experiments confirmed that miR-199a-3p decreased the expression of DDR1 via binding to DDR1 mRNA. In ovarian cancer cells, the miR-199a promoter was hypermethylated, but not in normal cells. Knockdown of DNMT3A increased miR-199a-3p expression and attenuated the expression of DDR1 in ovarian cancer cells, while overexpression of miR-199a-3p impaired the migratory, invasive and tumorigenic capabilities of ovarian cancer cells as well as enhanced cisplatin resistance through inhibiting DDR1 expression.

Using miRNA microarray analyses, Zhao *et al*.^[126]^ demonstrated that upregulation of miR-224-5p was associated with platinum-based chemo-resistance in ovarian cancer patients. They identified the protein kinase Cδ gene as one of the targets of miR-224-5p in mediating resistance to cisplatin in ovarian cancer patients. These findings indicate that miR-224-5p and protein kinase Cδ can serve as predictors and prognostic biomarkers for ovarian papillary serous cancer patient response to overall disease-specific survival.

The Notch receptor family plays an important role in cell differentiation, organ development and tumorigenesis, tumor progression, invasion and metastasis. The activation of the Notch signaling pathway can both accelerate and restrain tumorigenesis, depending on the cell environment^[50]^. Zhou *et al*.^[127]^ demonstrated that ectopically expressed miR-449a increased cisplatin sensitivity in cisplatin-resistant ovarian cell lines through targeting Notch1 transcripts, inhibited cell proliferation and promoted apoptosis. These findings were confirmed with *in vitro* experiments. When BALB/c nude mice were injected intraperitoneally with cisplatin-resistant ovarian cancer cells transfected with miR-449a, they exhibited enhanced cisplatin sensitivity *in vivo*.

Based on the interplay between miR-483-3p and protein kinase Cδ, Arrighetti *et al*.^[128]^ explained the mechanism of resistance to cisplatin. They observed that miR-483-3p usually involved in apoptosis and cell proliferation was up-regulated in cisplatin resistant ovarian cancer cells. This up-regulation of miR-483-3p and possibly its binding to protein kinase Cδ mRNA interfered with the proliferation of resistant ovarian cancer cells, thus, protecting them from DNA damage induced by platinum compounds.

MiR-509-3p has been reported to sensitize ovarian cancer cells to cisplatin treatment by targeting multiple anti-apoptosis genes including BCL2^[129,130]^. Niu *et al*.^[131]^ compared the expression profiles of miRNAs between three pairs of platinum-resistant and platinum-sensitive ovarian tissues and found that miR-509-3p was significantly down-regulated in cisplatin-resistant ovarian cancer tissues. Functional studies demonstrated that miR-509-3p inhibitor decreased cell response to cisplatin and promoted cell survival in ovarian cancer cells. In this process, miR-509-3p regulated the expression of Golgi phosphoprotein-3 (GOLPH3) and wntless Wnt ligand secretion mediator (WLS).

Previously, Zhang *et al*.^[132]^ detected that miR-520g contributes to tumor progression and cisplatin resistance by post-transcriptionally downregulating its target mRNA of death-associated protein kinase 2 (DAPK2). MiR-520g expression gradually increased across normal, benign, borderline and ovarian cancer tissues. High miR-520g levels promoted tumor progression and chemo-resistance to cisplatin, and reduced survival in ovarian cancer patients. DAPK2 overexpression or miR-520g knockdown reduced ovarian cancer cell proliferation, invasion, wound healing and chemo-resistance.

Ovarian cancer stem cells are involved in tumor growth, metastasis and recurrence. The main characteristics of this subpopulation of cancer cells are their uncontrolled proliferation, high invasiveness and resistance to the current platinum-based therapies^[133]^. Using a quantitative PCR array, Wei *et al*.^[134]^ demonstrated that miR-551b was upregulated in ovarian cancer stem cells and that its levels correlated with the pathological grades. In vitro experiments indicated that miR-551b inhibited the transcription factor forkhead box O3 and RING finger, B-box and coiled-coil domain protein TRIM31 (tripartite motif), promoted proliferation, invasion and chemo-resistance of ovarian cancer cells and cancer stem cells. Accordingly, in mouse xenograft models, the inhibition of miR-551b significantly increased the susceptibility of ovarian cancer cells to cisplatin and prolonged the survival of the host mice.

Excision repair crossing-complementing group 2 (ERCC2), also called xeroderma pigmentosum complementary group D (XPD), plays a crucial role in the nucleotide excision repair pathway. In concert with XPA, ERCCR2 verifies the presence of a relevant base lesion by scanning a DNA strand in the 5'-3' direction, so ensuring the accurate removal of the lesion from the genome^[135]^. In this regard, Zhao *et al*.^[136]^ examined the function of miR-770-5p which targets ERCCR2 in cisplatin chemotherapy resistance in ovarian cancer patients. MiR-770-5p expression was reduced in these patients. Overexpression of miR-770-5p reduced survival in chemo-resistant cell lines after cisplatin treatment by downregulating ERCC2. A comet assay confirmed that the restoration of cisplatin chemo-sensitivity was due to the inhibition of DNA repair.

The insulin-like growth factor-1 receptor (IGF-1R) is expressed on most transformed cells, where it has anti-apoptotic, cell-survival and transforming activities. Its activation is a hallmark for tumor initiation and progression^[137]^. Zhang *et al*.^[138]^ investigated the effect of miR-1294 on platinum-resistant ovarian cancer and documented that miR-1294 dysregulation manipulated ovarian cancer cisplatin resistance through regulating IGF1R. Knockdown of IGF1R decreased cell proliferation, migration, invasion and EMT of cisplatin-resistant cells. Overexpression of miR-1294 inhibited cisplatin resistance, suggesting that epigenetic regulation of IGF1R by miR-1294 was essential for cisplatin resistance.

### Carboplatin resistance

Our previous data indicated the relevance of dysregulated plasma miR-146a in different breast cancer subtypes, suggesting its potential role in breast cancer biology and tumor progression^[139]^. Wilczyński *et al*.^[140]^ compared miR-146a expression levels in primary tumors and omental metastases of 48 patients who had undergone surgery of advanced ovarian serous cancer. The miR-146a levels in primary tumors were elevated compared with normal ovary tissues and metastases. There was a negative correlation between miR-146a expression in primary tumors and serum amounts of cancer antigen 125 (CA125). Decreased miR-146a expression was associated with a shorter overall and progression-free survival, most notably with carboplatin resistance of metastases.

Using real-time qPCR miRNA OpenArrays, Benson *et al*.^[141]^ measured miRNA concentrations in plasma samples from 14 patients with platinum-resistant, recurrent ovarian cancer enrolled in a phase II clinical trial that were treated with a low dose of the hypomethylating agent decitabine followed by carboplatin. Ten miRNAs (miR-193a-5p, miR-375, miR-339-3p, miR-340-5p, miR-532-3p, miR-133a-3p, miR-25-3p, miR-10a-5p, miR-616-5p and miR-148b-5p) displayed multi-fold changes in concentrations in recurrent platinum resistant ovarian cancer patients that were associated with a response to decitabine followed by carboplatin chemotherapy. In addition, circulating miR-148b-5p concentrations were associated with progression-free survival and may represent a novel biomarker for therapeutic response to this chemotherapy regimen in patients with recurrent, drug-resistant ovarian cancer.

### Cis- and carboplatin resistance

MiRNA can be released by apoptotic and necrotic cells into the blood circulation, but they can also be actively secreted in extracellular vesicles (EVs). EVs are thought to play an important role in cell-to-cell communication^[142]^. In this regard, Kuhlmann *et al*.^[143]^ designed an integrated NGS-based workflow for analyzing the signature of EV-associated miRNAs in the plasma of platinum-resistant ovarian cancer patients. They found a panel of EV-associated miRNAs, such as miR-181a, miR-1908, miR-21, miR-486 and miR-223, which were differentially abundant in the plasma of platinum-resistant patients.

BRCA1 and BRCA2 play an important role in the homologous recombination DNA repair system. Cells harboring mutations of BRCA1/BRCA2 are especially sensitive to platinum^[144]^. Furthermore, the Ku heterodimer consisting of two subunits (Ku70 and Ku80) plays a central role as an initial DNA end binding factor in the classical non-homologous end joining pathway^[145]^. Choi *et al*.^[146]^ detected a resistance mechanism by which miR-622 induced cis- and carboplatin resistance in BRCA1 mutant high-grade serous ovarian carcinomas by targeting the Ku complex and restoring homologous recombination mediated double strand break repair. MiR-622 inversely correlated with Ku expression during the cell cycle, suppressed non-homologous end-joining, but facilitated homologous recombination mediated double strand break repair in the S phase. Notably, a high expression of miR-622 in BRCA1-deficient high-grade serous ovarian carcinomas correlated with worse outcome after platinum chemotherapy.

## Conclusion

Despite recent advances in treatment regimens, ovarian cancer remains one of the most deadly diseases because of its development of drug resistance. Due to the high number of relapsed ovarian cancer patients, new therapy options for platinum resistant disease are needed. The cytotoxicity of platinum is based on the formation of DNA adducts, including DNA-protein cross-links, DNA monoadducts and interstrand DNA cross-links, activating DNA damage and consequently, the cell death pathway. However, the events leading to platinum resistance are not well understood. Besides genetic alterations, changes in epigenetic regulation may contribute to this resistance. In particular, epigenetically silenced tumor suppressor genes involved in apoptosis, DNA repair and the cell cycle may be the main reasons for drug resistance. As shown by previous studies and described above, among other factors, BRCA1, BRCA2, MLH1, p53 and p21 contribute to platinum resistance via DNA damage and repair, while p21, RASSF1, Bax and p53 contribute via apoptosis. The most relevant signaling pathways and processes participating in platinum resistance include Wnt, PI3K/Akt, Notch, NF-κB and EMT. As specified above, epigenetic therapies appear to be promising therapy strategies. Combined administration of DNMT and HDAC inhibitors may re-express silenced tumor suppressor genes. Moreover, changes in the methylation profiles of ovarian cancer have led to the testing of new combination treatment regimes. Therapeutics to inhibit DNMT, including azacitidine and decitabine, were successfully developed and approved for treatment. In 2010, Fang *et al*.^[79]^ were the first to assess decitabine at repeated low doses to reduce DNA methylation and re-instate cisplatin sensitivity in a Phase 1 clinical trial of HGSOC patients. Other therapeutic agents that target methylation include SGI-110 as a nucleoside analogue and valproic acid, and seem to be beneficial in the treatment of diverse cancer types.

Further epigenetic targets could be miRNAs that are also involved in tumor suppressor silencing. Accordingly, miRNAs are attractive candidates for developing a new class of drugs that specifically target miRNA pathways. For example, promising targets to date are miR-622^[146]^ that targets the Ku pathway and miR-484 that targets both VEGFB and VEGFR2 pathways as well as tumor vasculature^[187]^. As reviewed above, many other miRNAs have also been associated with resistance to cis- and carboplatin in ovarian cancer. Therefore, determining which miRNAs are the best for miRNA targeted therapy development will be a challenge. In this regard, several mechanisms to target miRNAs are currently in development for cancer treatment. Down-regulation of target oncogenes by re-expression of tumor suppressor miRNAs, or re-expression of tumor suppressor genes by silencing of oncogenic miRNAs is anticipated to sensitize tumor to platinum treatment. Restoring and blocking miRNA function may be performed by replacement of tumor suppressor miRNAs with either synthetic or viral vectors encoded for miRNA mimics, or by antisense-mediated inhibition of oncogenic miRNAs, respectively. The above studies provide promising results to re-sensitize both ovarian cancer cell lines and animal models to platinum therapy, so laying the basis for effective epigenetic drugs in combination with platinum-based agents. In future, cis- or carboplatin therapies combined with epigenetic drugs may shed light on the potential of personalized treatment modalities to overcome resistance in women with recurrent ovarian cancer.

## Declarations

### Copyright

© The Author(s) 2019.
